# Quantum Simulation Study of Ultrascaled Label-Free DNA Sensors Based on Sub-10 nm Dielectric-Modulated TMD FETs: Sensitivity Enhancement Through Downscaling

**DOI:** 10.3390/mi16060690

**Published:** 2025-06-08

**Authors:** Khalil Tamersit, Abdellah Kouzou, José Rodriguez, Mohamed Abdelrahem

**Affiliations:** 1 National School of Nanoscience and Nanotechnology, Abdelhafid Ihaddaden Science and Technology Hub, Sidi Abdellah, Algiers 16000, Algeria; 2 Laboratory of Inverse Problems, Modeling, Information and Systems (PIMIS), Université 8 Mai 1945 Guelma, Guelma 24000, Algeria; 3 Applied Automation and Industrial Diagnosis Laboratory (LAADI), Faculty of Science and Technology, Djelfa University, Djelfa 17000, Algeria; kouzouabdellah@ieee.org; 4 High-Power Converter Systems (HLU), Technical University of Munich (TUM), 80333 Munich, Germany; 5 Center for Energy Transition, Universidad San Sebastián, Santiago 8420524, Chile; jose.rodriguezp@uss.cl; 6 Electrical Engineering Department, Faculty of Engineering, Assiut University, Assiut 71516, Egypt

**Keywords:** nanobiosensor, deoxyribonucleic acid (DNA), downscaling, dielectric modulation, field-effect transistor (FET), label-free detection, NEGF simulation, transition metal dichalcogenide (TMD)

## Abstract

In this article, the role of downscaling in boosting the sensitivity of a novel label-free DNA sensor based on sub-10 nm dielectric-modulated transition metal dichalcogenide field-effect transistors (DM-TMD FET) is presented through a quantum simulation approach. The computational method is based on self-consistently solving the quantum transport equation coupled with electrostatics under ballistic transport conditions. The concept of dielectric modulation was employed as a label-free biosensing mechanism for detecting neutral DNA molecules. The computational investigation is exhaustive, encompassing the band profile, charge density, current spectrum, local density of states, drain current, threshold voltage behavior, sensitivity, and subthreshold swing. Four TMD materials were considered as the channel material, namely, MoS_2_, MoSe_2_, MoTe_2_, and WS_2_. The investigation of the scaling capability of the proposed label-free gate-all-around DM-TMDFET-based biosensor showed that gate downscaling is a valuable approach not only for producing small biosensors but also for obtaining high biosensing performance. Furthermore, we found that reducing the device size from 12 nm to 9 nm yields only a moderate improvement in sensitivity, whereas a more aggressive downscaling to 6 nm leads to a significant enhancement in sensitivity, primarily due to pronounced short-channel effects. The obtained results have significant technological implications, showing that miniaturization enhances the sensitivity of the proposed nanobiosensor.

## 1. Introduction

The rapid advancement in the field of biosensing has significantly transformed the landscape of diagnostic technologies, particularly in the detection of biological molecules such as viruses, bacteria, glucose, streptavidin to biotin, and particularly deoxyribonucleic acid (DNA) [1,2,3]. Among the various approaches developed, label-free DNA sensors have garnered considerable attention due to their ability to detect DNA molecules without the need for additional labeling agents, thereby simplifying the detection process, reducing the overall cost, and improving sensitivity [4,5,6,7,8]. Traditional DNA sensors often rely on labeling techniques that can introduce complexity and cost into the biosensing approach. In contrast, label-free sensors offer a more straightforward, efficient, and reliable alternative, making them highly desirable for various applications, including medical diagnostics, ecological surveillance, forensic analysis, drug discovery and development, quality control and food safety, biotechnology, and relevant research [1,2,3,4,5,6,7,8,9,10].

Emerging 2D nanomaterials have played a pivotal role in the evolution of label-free DNA sensors [11,12,13]. Among these materials, transition metal dichalcogenides (TMDs) have gained attention as highly promising materials because of their unique electrical, optical, and mechanical properties [14]. TMDs, such as MoS_2_, MoSe_2_, and WS_2_, offer high surface-to-volume ratios, excellent conductivity, and tunable bandgaps, making them ideal for applications in nanoelectronics and biosensing [14,15,16]. The use of TMDs in nanoscale FETs has opened up new opportunities for advancing highly sensitive biosensors. When integrated into FETs, TMDs enable the detection of biomolecules at extremely low concentrations, enhancing the performance of label-free DNA sensors [17,18,19].

Dielectric-modulated field-effect transistors (DM-FETs) represent a significant advancement in the design of biosensors [20,21,22]. By modulating the dielectric environment around the FET channel, these devices can identify the presence of biomolecules, such as DNA, RNA, and various antibodies and antigens by measuring changes in electrical properties and metrics such as the threshold voltage, drain current, transconductance, current ratio, etc. The dielectric modulation technique leverages the information about the dielectric constant increment induced by the interaction between the target molecules and the probes to induce a measurable signal, thereby allowing for label-free bio-detection [20,21,22,23,24,25,26,27,28]. A practical example is the development of a point-of-care testing platform featuring a DM FET array and its readout circuitry for the high-performance detection of avian influenza [25]. The integration of emerging 2D materials (e.g., TMD nanomaterials) with DM-FETs can lay the foundation for the development of high-performance nanoscale biosensors that can revolutionize the field of biosensing.

The development of cutting-edge FET-based label-free biosensors requires meeting several key criteria, including high sensitivity, selectivity, rapid response times, and scalability [29,30,31,32]. The ability to detect extremely low concentrations of biomolecules is critical for the early diagnosis and monitoring of diseases. Selectivity ensures that the sensor responds only to the target molecules, minimizing false positives and negatives. A rapid response time is essential for real-time monitoring applications, while scalability is important for the mass production of nanosensors at low costs with possible adoption of cartridge configuration to avoid any cross-contamination issues [20,21,22,23,24,25,26,27,28,29,30,31,32,33,34]. Achieving most of these requirements, which form the basis of this work, often involves the careful design of the nanoscale FET structure, the selection of appropriate nanomaterials, the adoption of appropriate DNA sensing principle, finding the optimal working regime, and the optimization of the geometrical, physical, and electrical nano-biosensor parameters.

In this context, the present investigation explores the role of downscaling in enhancing the sensitivity of a novel label-free DNA nano-biosensor utilizing dielectric-modulated transition metal dichalcogenide field-effect transistors (DM-TMDFETs) endowed with all-around gates. Using a comprehensive quantum simulation approach, we examine how the DNA binding-induced increase in the biocavity’s dielectric constant influences various sensor characteristics, including the band profile, charge density, current spectrum, threshold voltage behavior, and sensitivity. Our results indicate that the downscaling of DM-TMDFETs not only enables the development of highly compact nanoscale biosensors but also significantly boosts their sensitivity, making them capable of detecting DNA molecules with dimensions comparable to the nanosensor itself. In addition, we found that the sensitivity may show only a slight enhancement when the device is scaled down from 12 nm to 9 nm; however, a more aggressive reduction to 6 nm results in a significant increase in sensitivity, which is mainly attributed to severe short-channel effects. This study highlights the potential of TMD-based DM-FETs as a powerful foundation for the development of next-generation label-free DNA sensors, with significant implications for the broader field of biosensing.

The rest of this article is carefully arranged as follows. Section 2 introduces the structure of the FET-based DNA nanosensor, along with its working and sensing principles. This section delves into the underlying mechanisms that enable label-free DNA sensing at the nanoscale, with a particular focus on the role of dielectric modulation concept. Section 3 outlines the atomistic simulation method used in this numerical study, detailing the self-consistent coupling of the Non-Equilibrium Green’s Function (NEGF) formalism with the Poisson equation. The computational models and methods, as well as the main equations used to simulate the proposed TMD FET-based label-free DNA nanosensor, are thoroughly discussed. Section 4 presents and analyzes the numerical results, exploring the impact of downscaling on sensor performance, including aspects such as the band profile, charge density, current spectrum, and sensitivity. Finally, Section 5 provides the conclusion, summarizing the key findings of the study and offering insights into the potential applications of the proposed nanosensor, along with suggestions for future research directions.

## 2. Nanobiosensor Structure and Principle of DM-Based Label-Free DNA Detection

**Figure 1:**
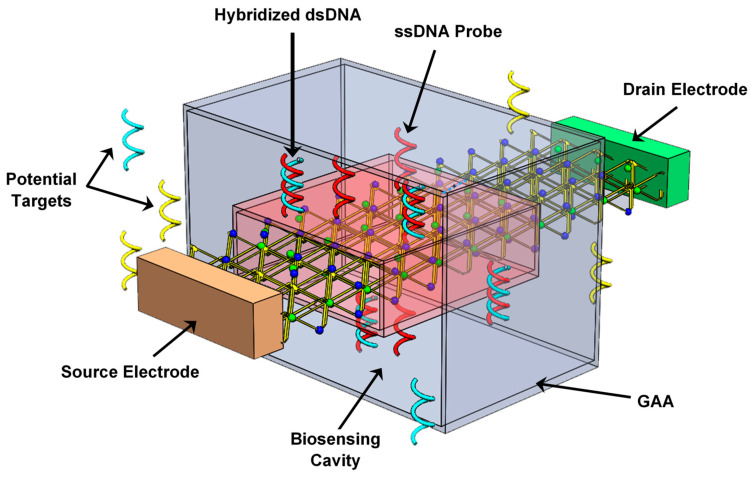
Three-dimensional structure of the proposed label-free DNA nanosensor based on an ultra-scaled gate-all-around transition metal dichalcogenide field-effect transistor.

Figure 1 shows the three-dimensional structure of the proposed GAA TMDFET-based label-free DNA nanosensor. The biosensing cavity is clearly visible beneath the gate, with thickness t_CAV_, which can experimentally be created using a sacrificial layer followed by wet etching techniques [20,21,22,23,24]. An ultra-thin SiO_2_ oxide layer is retained on the channel material to protect the TMD channel from any unintentional impurities. The TMD channel features an n-i-n doping profile with a highly n-type doped source and drain reservoirs with L_S(D)_ lengths. Some single-stranded DNA (ssDNA) probes are illustrated as being attached within the biosensing cavity, which can be achieved using self-assembled monolayer techniques [23,24,32]. Additionally, the figure shows some double-stranded DNA (dsDNA) molecules, indicating the interaction between the ssDNA probes and the DNA targets, resulting in the formation of double-stranded helix structures where two complementary strands are aligned according to the well-known base pairing: Adenine (A) with Thymine (T) and Cytosine (C) with Guanine (G) [32,33,34,35].

**Figure 2:**
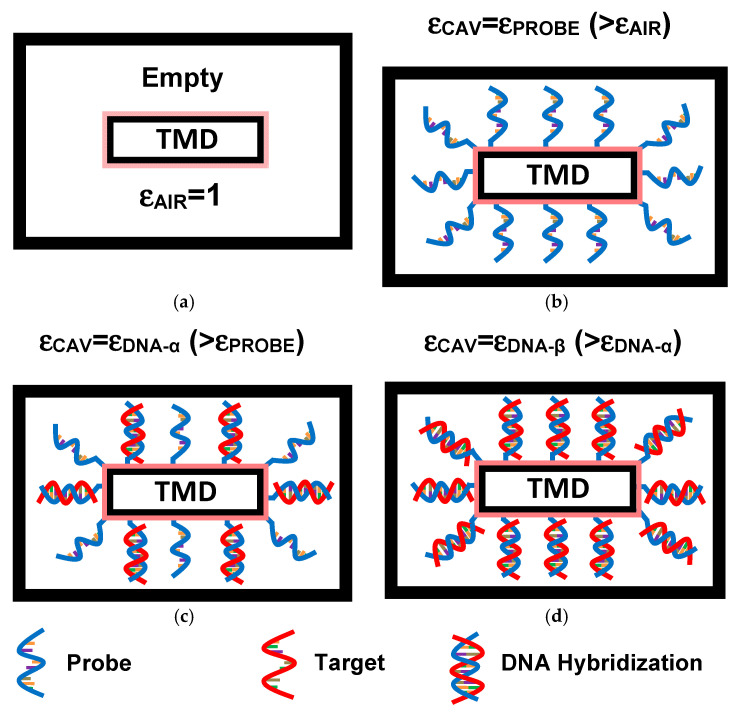
Illustration of the process of label-free DNA detection utilizing the dielectric modulation concept within the proposed TMDFET-based nanobiosensor. (**a**) An empty open biosensing cavity, (**b**) a biosensing cavity containing ssDNA probes, (**c**) a biosensing cavity with ssDNA probes and hybridized dsDNA molecules, and (**d**) a biosensing cavity filled with a higher density of hybridized DNA molecules.

The label-free DNA sensing mechanism operates through dielectric modulation caused by neutral DNA molecules within the cavity created beneath the gate-all-around (GAA) structure [23,24,32,33,34]. Figure 2 illustrates cross-sectional views perpendicular to the gate-all-around, highlighting the key bio-events involved in the label-free DNA sensing process. In Figure 2a, the cavity of the proposed GAA TMDFET-based label-free DNA nanosensor is empty, with a dielectric constant equivalent to that of air (i.e., ε_AIR_ = 1). This initial state can be characterized by a reference threshold voltage, measurable using an appropriate readout circuit [23]. Subsequently, single-stranded DNA molecules are introduced into the cavity beneath the GAA to establish the selectivity of the proposed DNA nanosensor for specific DNA targets [23], as depicted in Figure 2b. Given the presence of neutral DNA molecules, the insertion of ssDNA probes into the biocavity raises the dielectric constant, resulting in an alteration in drain current and a shift in threshold voltage due to the change in the device electrostatics. At this stage, the label-free DNA sensor is prepared to detect target DNA via the hybridization process [23,24]. Figure 2c shows the formation of double-stranded DNA resulting from the hybridization of ssDNA probes with ssDNA targets, marking a successful biodetection event [23]. According to the dielectric modulation concept [23,24], the presence of dsDNA molecules causes a further increase in the dielectric constant compared to the scenario with only ssDNA probes, as shown in Figure 2b. The difference between the recorded threshold voltages before and after hybridization provides valuable information about the bio-events occurring within the nanogap, such as DNA mismatches, hybridization, de-hybridization, relevant concentrations, and more [23]. Figure 2d depicts a third increase in the cavity’s dielectric constant due to the higher density of hybridized dsDNA molecules [23]. It is important to highlight that, since the dielectric modulation phenomenon occurs specifically during the introduction of ssDNA probes and subsequent DNA hybridization, it inherently reflects the hybridization process, a highly selective molecular recognition mechanism. As a result, the biosensor naturally exhibits high selectivity and strong anti-interference capability. Regarding the sensing parameters, other sensing metrics, such as the on-state current, off-state current, current ratio, subthreshold swing, conductance, and transconductance, can also be used to monitor bio-events, in addition to the shift in threshold voltage, which is the commonly used biometric in dielectric modulated FET-based biosensor [20,21,22,23,24,25,26,27,28].

## 3. Quantum Simulation Approach

The NEGF simulation method offers significant benefits by accurately capturing a wide range of quantum transport phenomena and electrostatic features, which play an essential role in nanoscale field-effect transistors, including FET-based sensors and biosensors [36,37]. It allows for the precise modeling of charge transport and 2D/3D potential distribution at the ultrascaled level, ensuring that the complex behaviors of charge and electrostatic effects are fully considered, which is crucial for assessing cutting-edge electron nanodevices [36,37,38,39,40,41,42,43], including the ultrascaled DM-FET-based biosensor under investigation. The evaluation of the proposed gate-all-around TMD FET-based DNA nanosensor is conducted using a quantum simulation approach. This method involves a self-consistent solution of the Poisson equation in conjunction with the Schrödinger equations using the NEGF formalism [44,45,46,47]. Given the ultrascaled channel length (i.e., sub-10 nm), ballistic transport is assumed, with scattering mechanisms being disregarded (Σ_SCAT_ = 0) [46]. It is worth noting that ballisticity has been found to be relatively dominant in sub-8 nm MoS_2_ FETs, and the current ratios, with and without scattering, remain nearly identical as the gate length decreases [44]. The TMD monolayers are modeled computationally using an effective mass-based Hamiltonian, *H*, which is integrated into the calculation of the Green’s function *G*, as described in [37,38,48]:

(1) $$G(E)={\left[(E+i0^{+})I-H-\sum_{S}-\sum_{D}\right]}^{-1}$$

where *E* is the energy, *I* is the identity matrix, 0^+^ denotes an infinitesimal positive value, and Σ*_S_*_(*D*)_ corresponds to the self-energy for the source (drain) contact [45]. The local density of states *D_S_*_(*D*)_ can then be calculated as [37]:

(2) $$D_{S(D)}=G\Gamma_{S(D)}G^{†}$$

where $\Gamma_{S(D)}=i(\Sigma_{S(D)}-\Sigma_{S(D)}^{†})$ is the broadening function associated with the source (drain) contact. It is important to note that the transmission can be calculated as [46]:

*T*(*E*) = *Trace*[Γ*_S_G*Γ*_D_G*^†^] (3)

In terms of electrostatics, the Poisson equation was solved, which is expressed as:

−∇ · (*ε_r_*∇ *V*) = *ρ*/*ε*_0_ (4)

where *ε_r_* is the relative permittivity of the materials, *V* is the electrostatic potential, *ρ* represents the charge density, and *ε*_0_ is the permittivity of free space. In the context of DNA sensing using the dielectric modulation concept, the dielectric constant is assigned as *ε_DNA_* in the region occupied by DNA molecules, *ε_AIR_* in the area filled with air, and *ε_OX_* in the oxide region [32,33,34]. The assumption of a homogeneously filled biosensing cavity is justified by the sub-10 nm scale of the sensing region, comparable to DNA dimensions, promoting uniform occupation. Additionally, the open cavity geometry enhances molecular diffusion and stable attachment, supporting the use of an effective dielectric constant. Note that the considered range of the dielectric constant (i.e., from ε_AIR_ = 1 to ε_DNA_ = 8) is commonly adopted in the literature, both computationally and experimentally [23,24,32,33,34]. It is worth pointing out that the dielectric inhomogeneity can be a matter for further investigations. The Neumann boundary condition is applied to all external interfaces, except at the GAA nodes, where the Dirichlet boundary condition is used. Once the self-consistent NEGF-Poisson system reaches convergence, the drain current can be determined as described in [46].

It is worth noting that the NEGF-based quantum simulation approach serves as a robust predictive tool for designing and optimizing FETs, including FET-based sensors and biosensors, even when experimental data are unavailable. By self-consistently solving the Poisson and Schrödinger equations, this method accurately captures the behavior of nanoscale devices, factoring in quantum effects that are crucial at sub-10 nm dimensions. This approach allows researchers to forecast device performance, fine-tune material and structural parameters, and investigate novel sensing mechanisms prior to fabrication, making it essential for advancing next-generation FET-based biosensors with exceptional sensitivity and scalability. For more detailed information on the NEGF simulation approach, we refer to some relevant computational studies [36,37,38,39,40,41,42,43,44,45,46,47,48,49,50].

## 4. Results and Discussion

**Figure 3:**
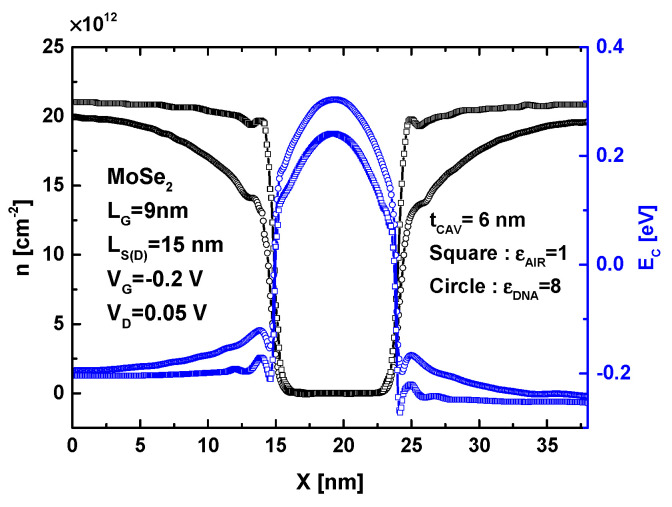
Band profile and charge density of MoSe_2_ FET-based biosensor before and after DNA introduction.

Figure 3 illustrates the behavior of the proposed DNA sensor before and after the introduction of DNA molecules, focusing on the band profile and charge density. A sub-10 nm gate length, L_G_, and low drain-to-source voltage (V_DS_ = 0.05 V) were considered to ensure the features of small size and low power consumption, which are essential prerequisites for cutting-edge nanobiosensors. Note that the gate-to-source voltage (V_GS_) was chosen to be in the subthreshold regime. The simulation parameters are clearly displayed as an inset in the same figure. It is clearly observed that the DNA-induced increase in the cavity’s dielectric constant (i.e., from ε_AIR_ = 1 to ε_DNA_ = 8) results in a significant elevation of the potential barrier. Additionally, an elevation in the band profile is observed at the source and drain reservoir regions, where the modulation is most significant near the electrostatic gating region. This DNA-induced increase in the potential profile decreases as it approaches the source and drain electrodes. Furthermore, the same figure shows that the DNA-induced increase in the cavity’s dielectric constant leads to a decrease in charge density, particularly at the sides of the main electrostatic gating region. The behavior in terms of the band profile and charge density suggests a decrease in the subthreshold drain current.

**Figure 4:**
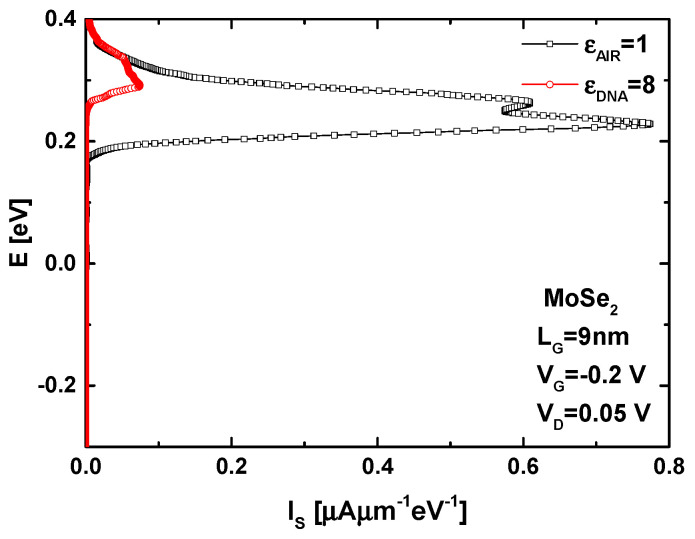
Current spectrum of the GAA MoSe2 FET-based DNA nanosensor before and after the introduction of DNA molecules.

Figure 4 shows the current spectrum of the proposed MoSe_2_ FET-based DNA nanosensor under the same biasing conditions (i.e., the subthreshold domain), the DNA-induced increase in the dielectric constant (i.e., from ε_AIR_ = 1 to ε_DNA_ = 8), and the gate length, as in Figure 3. As expected, the biomolecule-induced elevation in the potential barrier results in a reduced thermionic emission component due to the opposition of the potential barrier, leading to a significant decrease in the current spectrum, as clearly shown. In both cases, a slight direct source-to-drain tunneling (DSDT) is recorded through the top of the potential barrier due to its narrow width. The DSDT-linked current spectrum then tends towards a very weak current. This is attributed to the opposition of the potential barrier and the high effective mass of carriers [51,52], in contrast to carbon FETs [53,54].

**Figure 5:**
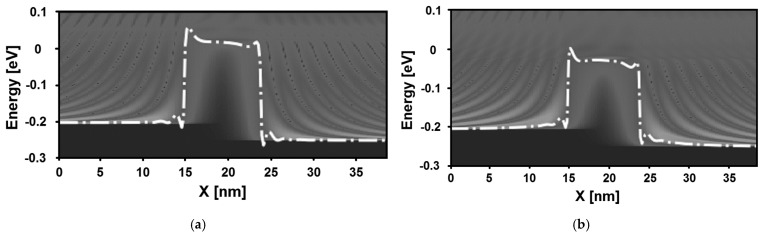
Local density of states of the MoSe_2_ FET-based DNA sensor, (**a**) with an empty cavity and (**b**) with a cavity filled with specific DNA molecules corresponding to ε_DNA_ = 8 at V_DS_ = 0.05 V and V_GS_ = 0.5 V.

Figure 5 shows the local density of states (LDOS) of the proposed DM MoSe_2_ nanoFET-based DNA sensor, comparing the cases of an empty cavity and a cavity filled with DNA molecules, corresponding to a specified dielectric constant of ε_DNA_ = 8. Note that the DNA nanosensor is biased in the above-threshold regime. In both figures, oscillation patterns, attributed to quantum mechanical reflections, are evident. By comparing the two figures, it is clear that the height of the potential barrier is reduced by the DNA-induced increase in the dielectric constant. This behavior leads to an increase in thermionic emissions, as shown by the lighter spectrum over the potential barrier in Figure 5b compared to Figure 5a, where the spectrum appears darker. Based on the observed behavior in terms of the potential profile, charge density, current spectrum, and LDOS in both the subthreshold and above-threshold regimes, an improvement in the subthreshold swing is expected. This is logical, given that the dielectric constant of the nano-device increased from the unity of air to higher values.

**Figure 6:**
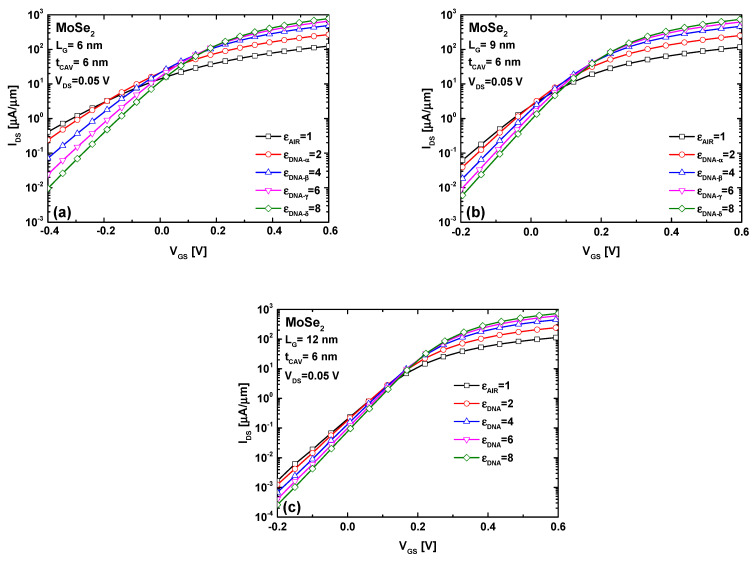
The I_DS_–V_GS_ characteristics of the dielectric modulated MoSe2 FET-based DNA nanosensor before and after DNA-induced dielectric increment, considering (**a**) L_G_ = 6 nm (**b**) L_G_ = 9 nm and (**c**) L_G_ = 12 nm.

Figure 6 shows the I_DS_–V_GS_ transfer characteristics of the MoSe_2_ FET-based biosensor, considering the DNA-induced increase in the cavity’s dielectric constant, as well as the effects of downscaling. For a fixed voltage in the subthreshold (above-threshold) regime, it is clear that, in all three gate length cases, the DNA-induced dielectric increase lowers the off-state current (subthreshold) and raises the on-state current (above-threshold). Inspecting the three figures, we can also observe an improvement or decrease in the subthreshold swing; this is expected, since the presence of DNA increases the coupling capacitance, thereby improving electrostatic control over the channel potential. By fixing the drain current for all cases and recording the corresponding gate voltage for each I–V property, we can see that this pseudo/biosensing threshold voltage shifts in a positive direction in all three gate length cases. It is worth noting that sensitivity is defined as the difference in this pseudo threshold voltage before and after DNA introduction (i.e., ∆V_TH_ = V_TH-DNA_ − V_TH-AIR_) [32].

It is important to emphasize that the same behavior was observed when using MoS_2_, MoTe_2_, and WS_2_ as channel materials. More importantly, by comparing the three figures to assess the impact of gate downscaling on the biosensing performance of the proposed TMD FET-based biosensor, we observe that decreasing the gate length makes the DNA-induced change in the biosensing threshold voltage more significant. Further inspection of Figure 6 reveals that relatively low I_ON_/I_OFF_ current ratios were recorded, which is attributed to the ultrascaled gate length, or, equivalently, the short-channel effects. However, we emphasize that the current ratio is not particularly important in our biosensing approach, as it is not relevant to digital applications and is not considered a key metric for biosensing.

**Figure 7:**
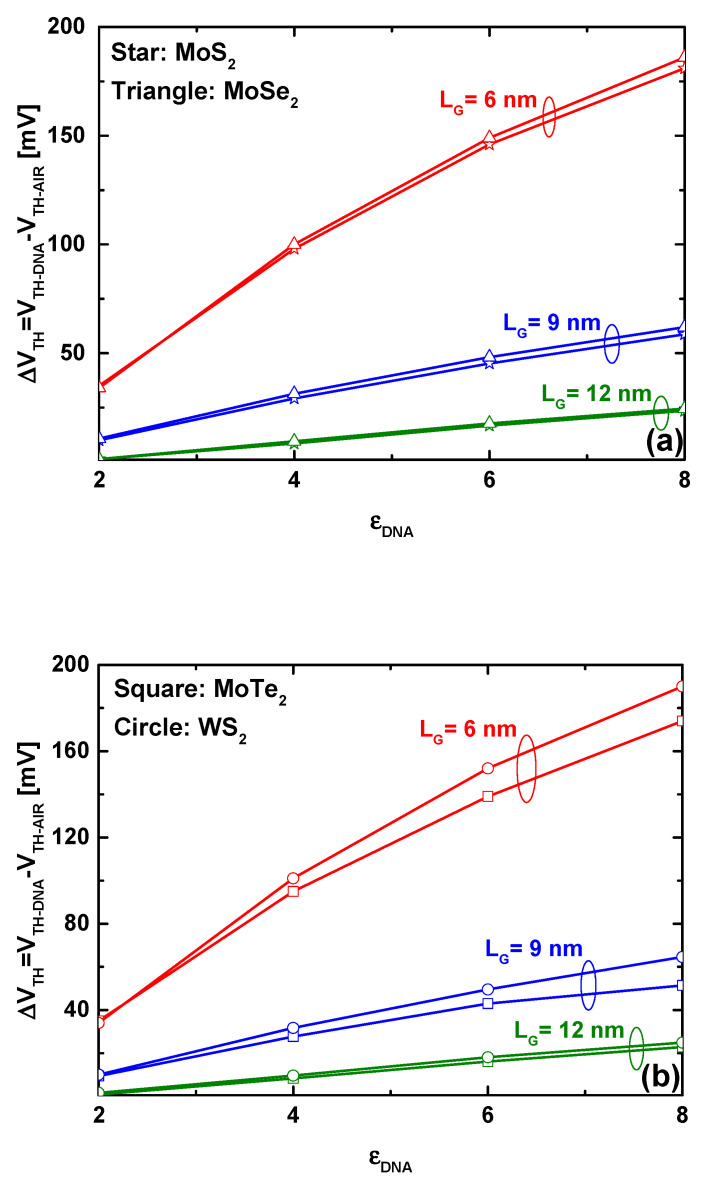
The sensitivity of the ultrascaled nanosheet FET-based DNA nanosensor as a function of the DNA dielectric constant for different gate lengths, considering (**a**) MoS_2_, MoSe_2_, and (**b**) MoTe_2_, WS_2_ as TMD channels. The figures illustrate how downscaling boosts the biosensor sensitivity.

Figure 7 illustrates the behavior of the proposed TMD FET-based biosensor in terms of sensitivity, considering the DNA-induced increase in the dielectric constant and different gate lengths, with four TMD nanomaterials as the channel: MoS_2_, MoSe_2_, MoTe_2_, and WS_2_. In both figures, it is evident that the DNA-induced increase in the dielectric constant results in a greater threshold voltage shift. As observed in the two figures, gate downscaling enhances sensitivity, with the TMD FET-based biosensor having a gate length of 6 nm, exhibiting high sensitivity. Note that the recorded behavior in terms of sensitivity downscaling suggests the potential to achieve ultra-high sensitivity at an aggressively scaled regime. Additionally, the ability to distinguish between different DNA dielectric constants is improved, which is highly beneficial from a readout circuitry perspective. The inspection of the two figures reveals that changing the channel TMD material does not significantly enhance biosensor sensitivity, as all biosensors exhibit almost the same sensitivity.

It is worth noting that the beneficial impact of gate length downscaling on sensitivity can be explained by the role of the coupling capacitance’s high dielectric constant in mitigating short-channel effects. In other words, the improvement in transfer characteristics becomes significant when the FET-based biosensor is subjected to severe short-channel effects. Similarly, the enhancement of electrostatic control in ultra-scaled FET-based biosensors through an increase in the gate dielectric constant is more substantial when the FET-based biosensor initially has poor electrostatic control (empty biosensing cavity) over channel transport.

**Figure 8:**
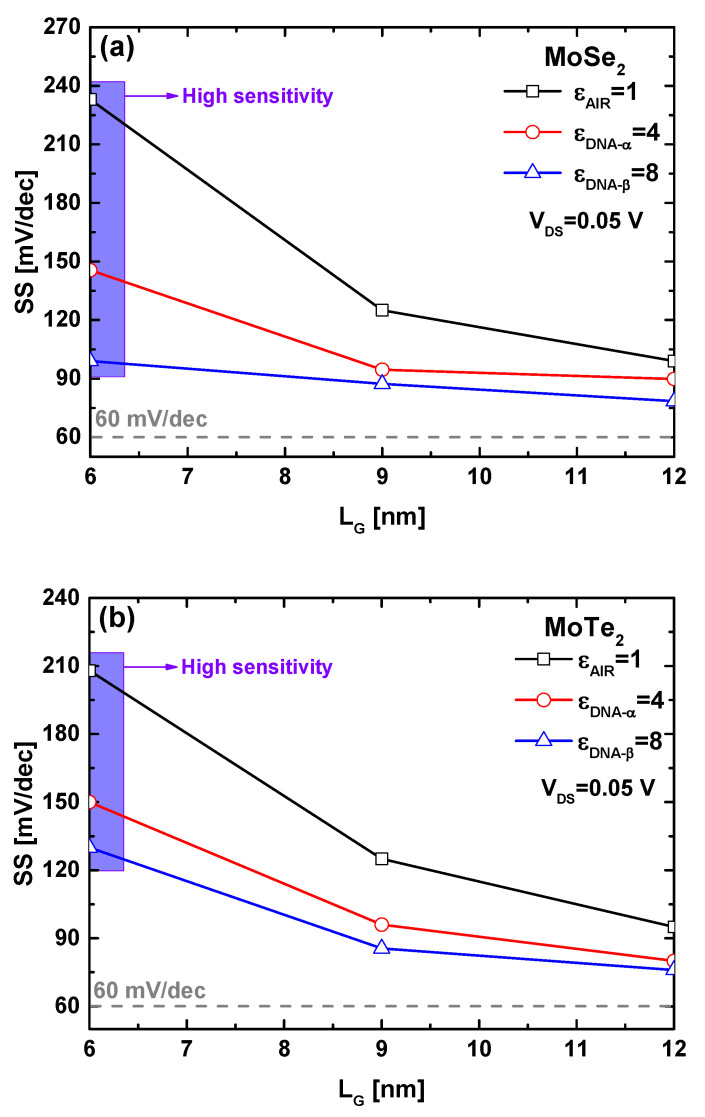
Subthreshold swing as a function of gate length, considering DNA-induced increments in the dielectric constant for (**a**) MoSe_2_ and (**b**) MoTe_2_ FET-based biosensors. The figures illustrate the rate of SS (ΔV_GS_/Δlog(I_DS_)) enhancement attributed to the increase in the DNA-induced dielectric constant.

To provide a clearer understanding of how the DNA-induced increase in the dielectric constant significantly impacts the drain current of the proposed biosensor in an aggressively scaled regime, we plotted the behavior of the MoSe_2_ and MoTe_2_ FET-based DNA nanosensor in terms of the subthreshold swing versus gate length downscaling for different DNA dielectric constants, as shown in Figure 8a,b, respectively. The typical behavior of a decrease in the swing factor with increasing gate length is clearly observed in all cases, regardless of variations in the channel material or the dielectric constant of the biosensing cavity. Inspecting the two figures, it is evident that a decrease in the subthreshold swing is recorded due to the increase in the cavity’s dielectric constant across the entire range of gate lengths. More importantly, this decrease in subthreshold swing, attributed to the DNA-induced dielectric constant increase, is significant at very short channel lengths and becomes less pronounced as the gate length increases. This indicates that the improvement in electrostatic gating via an increase in the gate dielectric constant is most substantial and explicitly evident at very short gate lengths, highlighting the beneficial role of gate downscaling in enhancing the biosensing performance of the proposed TMD nanosheet FET-based DNA nanosensors.

It is worth noting that the high selectivity of the proposed dielectric-modulated FET-based DNA sensor stems inherently from the dielectric modulation paradigm itself. This label-free detection mechanism relies on the hybridization of complementary single-stranded DNA (ssDNA) molecules—specifically, the pairing of Adenine (A) with Thymine (T), and Cytosine (C) with Guanine (G), which results in a localized increase in the dielectric constant within the sensing region [23]. This dielectric increment is significant only when perfect base pairing occurs between the probe and target ssDNA strands, thereby offering a highly selective response to matching DNA sequences [23,24]. In contrast, mismatched DNA sequences do not induce a noticeable shift in the threshold voltage, as they fail to produce substantial dielectric changes or electrostatic modulation. This distinct behavior underscores the inherent selectivity of dielectric-modulated biosensors, where the interplay of DNA hybridization, dielectric constant variation, and the threshold voltage shift ensures accurate molecular recognition.

It is important to note that the detection limit of the sensor, defined as the minimum detectable concentration of DNA (i.e., the density of hybridized double-stranded DNA molecules), is primarily influenced by the system’s noise characteristics and the resolution of the readout signal, particularly the signal-to-noise ratio. In this context, the design of the readout circuit becomes a key factor. A well-engineered, low-noise, and high-resolution readout circuit enhances the sensor’s ability to detect lower biomolecular concentrations by effectively distinguishing the signal from background noise [25].

From a manufacturing point of view, the fabrication of a DM TMD nanosheet gate-all-around FET for biosensing begins with the deposition of a monolayer or few-layer TMD (e.g., MoS_2_ or WS_2_) via chemical vapor deposition or exfoliation. The nanosheet is patterned to define the active channel using precision lithography and anisotropic etching. Instead of filling the cavity around the channel with a conventional gate oxide, an open cavity is deliberately formed (using the sacrificial layer etching techniques) to allow for dielectric modulation by biomolecules. Subsequent steps involve the deposition of inner spacers, the formation of conformal metal gates around the cavity, and the creation of low-resistance source/drain contacts, typically via edge-contact engineering [55]. Key challenges include maintaining the structural integrity of the monolayer TMD during etching, achieving high sensitivity to biomolecule-induced dielectric changes, and suppressing interface traps that degrade sensing accuracy. Future directions in the fabrication of such nanoFET-based biosensors can include optimizing cavity geometry, exploring functionalization techniques for selective detection, and minimizing contact resistance to enhance signal transduction.

Advanced optimizers based on bio-inspired algorithms [56,57,58] can be adopted in conjunction with the self-consistent quantum simulation approach (considering ballistic and diffusive transport conditions) to achieve optimal biosensing performance. The investigation of the orientation and distribution of attached neutral and charged DNA molecules in the field of biosensing could be an important avenue for further research [59,60,61]. Additionally, the use of ferroelectric-induced gate voltage amplification [62,63,64] can also be investigated to boost the performance of such ultrascaled FET-based biosensors.

## 5. Conclusions

This study demonstrates that downscaling plays a pivotal role in enhancing the sensitivity of label-free DNA nanosensors based on dielectric-modulated transition metal dichalcogenide field-effect transistors (DM-TMDFETs). Using comprehensive quantum simulations, we have shown that miniaturizing the gate in a gate-all-around configuration not only reduces the biosensor’s footprint but also significantly boosts its biosensing performance. The results reveal that the ultrascaling of DM-TMDFETs, when combined with dielectric modulation, enables the highly sensitive, label-free detection of neutral DNA molecules. Notably, changing the channel TMD material is not an effective strategy for significantly enhancing biosensor sensitivity, as it results in only small improvements. Furthermore, while downscaling the device dimensions from 12 nm to 9 nm results in moderate sensitivity improvements, a more aggressive scaling from 9 nm to 6 nm produces a pronounced and substantial enhancement in sensitivity due to severe short-channel effects. These results highlight that aggressively scaled nanodevices exhibit significant sensitivity improvements compared to moderate scaling and long channel devices. This work underscores device miniaturization as a key strategy for developing next-generation nanoscale biosensors and advancing DNA detection technologies. In particular, the proposed nanodevice, with dimensions comparable to those of DNA molecules, proves to be a strong candidate for high-performance biosensing in array-based configurations, with promising implications for biomedical diagnostics.

## Data Availability

The data that support the findings of this study are available from the first corresponding author (K.T.) upon reasonable request.
